# Recent advances in laryngoscopy in adults

**DOI:** 10.12688/f1000research.18544.1

**Published:** 2019-06-06

**Authors:** Matteo Parotto, Richard Cooper

**Affiliations:** 1 Department of Anesthesia, University of Toronto, Toronto, Canada; 2 Department of Anesthesia and Pain Management, Toronto General Hospital, Toronto, Canada; 3 Interdepartmental Division of Critical Care Medicine, University of Toronto, Toronto, Canada

**Keywords:** laryngoscopy, direct laryngoscopy, videolaryngoscopy, indirect laryngoscopy, intensive care, emergency care, operating room, anesthesiology, airway management

## Abstract

Recent advances in technology have made laryngoscopy less dependent upon a direct line of sight to achieve tracheal intubation. Whether these new devices are useful tools capable of increasing patient safety depends upon when and how they are used. We briefly consider the challenges in reviewing the emerging literature given the variety of devices, “experience” of the care providers, the clinical settings, and the definitions of outcome. We examine some of the limitations of conventional direct laryngoscopy, question the definitions we have used to define success, discuss the benefits of indirect (video) techniques, and review evidence pertaining to their use in the patients in the operating room, emergency department, and intensive care unit.

## Introduction

Laryngoscopy remains a key component of airway management in anesthesia, critical care, and emergency medicine. Advances in technology over the past two decades ^1^, including illumination by light-emitting diodes, liquid crystal display of the image, and complementary metal–oxide–semiconductor video chips, have provided significant improvements in devices available to clinicians, as carefully summarized in a previous F1000 review ^2^.

The availability of these new technologies has impacted clinical practice, and several recent studies have helped to focus our understanding of the role of these devices in routine and rescue airway management in various clinical settings. This article aims to provide a concise review of some relevant advances since the last F1000 review.

## Definitions of difficult laryngoscopy

Satisfaction with our intubation technique is largely a result of how we define success. We often describe intubation success in binary terms (success versus failure), irrespective of the duration of the effort, the number of attempts, the amount of force, the adjuncts required, and the laryngeal view obtained ^3^. This fails to identify degrees of difficulty. When we frame complications in terms of requiring intensive care unit (ICU) care, brain injury, death, or necessitating an emergency surgical airway, the incidence of such catastrophes is small, but the impact may be great and largely avoidable ^4^. If direct laryngoscopy (DL) fails to reveal the larynx in nearly 6% of seemingly anatomically normal patients ^5^, there is little room for complacency. Mild and moderate difficulties were encountered by experienced anesthesiologists in 38% and 8% of intubation attempts, respectively, and more than three attempts were required in 3% of patients ^6^. In emergency medicine and critical care, there is evidence that the number and severity of the complications increase with each additional laryngoscopy effort ^7, 8^. Although evidence that this applies to physiologically optimized elective surgical patients is currently lacking, it is an aspirational goal to aim for “first-attempt intubation success” in every patient encountered.

## Rescue after failed direct laryngoscopy

Aziz *et al*. published a retrospective comparative analysis from the Multicenter Perioperative Outcomes Group on the success rate for five commonly used devices to achieve successful tracheal intubation after failed DL ^9^. Their study involved seven academic centers and reported 1427 cases of failed DL in nearly 350,000 patients in whom tracheal intubation had been attempted. Subsequent intubation attempts involved five rescue techniques in sufficient numbers to permit analysis: (i) video laryngoscopy (VL), (ii) flexible bronchoscopic intubation, (iii) rescue with a supraglottic airway as a conduit for intubation, (iv) optical stylet, and (v) lighted stylet. Their data showed that, between 2004 and 2013, airway providers increasingly selected VL as the rescue technique of choice at the expense of all other techniques. Furthermore, VL yielded the highest intubation success rate ^9^. Importantly, in this retrospective study, patients were not randomly assigned and thus the groups may have been dissimilar and intubation was most likely attempted by using the device that the care providers felt most confident using. The skills of the laryngoscopists very likely varied, and no standardized training had been implemented. Finally, the authors noted that care providers often chose an alternative to DL after only one failed attempt. To the extent that these seven academic centers are reflective of anesthesiology practice in general, VL has progressively supplanted other rescue methods and appears to be accomplishing its intended purpose with a greater degree of success.

## Primary laryngoscopy technique

### In the operating room

A recent systematic review explored the question of whether VLs reduce intubation failure and complications compared with DL in adults ^10^. Lewis and colleagues found that VLs improved laryngeal views and reduced intubation difficulty scores, failed intubations and airway trauma, particularly among patients with a potentially difficult airway ^10^. When their hospital needed to replace their laryngoscope stock, the authors conducted a trial and subsequently elected to convert to routine VL throughout the operating rooms and ICU. They documented the changes in attitudes throughout their department during and following this change, noting that the initial reluctance gave way to virtually unanimous and enthusiastic support of the substitution ^11^.

A French group evaluated a new algorithm for elective airway management in the operating room ^12^. Patients presenting features predictive of a difficult (direct) laryngoscopy received an “enhanced management” strategy that used a channeled optical laryngoscope (Airtraq, Vygon, Écouen, France; the Airtraq is operationally similar to a VL but uses prisms rather than a video camera to provide its view) as a first step in conjunction with a long flexible angulated stylet (Frova Intubating Introducer, Cook Medical, Bloomington, IN, USA) and a flexible videoendoscope (aScope 3™, Ambu, Ballerup, Denmark) as the second and third steps, respectively. This approach prospectively studied 16,695 patients, achieving successful tracheal intubation of all patients with anticipated difficult laryngoscopy ^12^. The stratification also resulted in relatively few patients with poor laryngeal views, requiring multiple attempts, or experiencing oxygen desaturation. The study was conducted in a single site with a group of anesthesiologists well trained in the use of the Airtraq device. They identified two features that appeared to predict difficulty with intubation using the Airtraq device: limited mouth opening and reduced cervical spine mobility. It is unclear whether these results are applicable to non-channeled VLs or in centers with less experience using the Airtraq.

An earlier, single-center Italian study prospectively evaluated a VL-based airway management algorithm involving 6276 patients ^13^. Patients exceeding their defined threshold of an El-Ganzouri Risk Index ^14^ (EGRI) of more than 7—combining assessments of mouth opening, thyromental distance, Mallampati class, cervical range of motion, ability to perform mandibular protrusion, body weight, and prior history of difficult intubation ^15^—were managed with awake flexible endoscopic intubation. Intubation was attempted with a GlideScope™ if the EGRI was less than 7. The investigators excluded patients with a body mass index (BMI) of more than 30 kg/m ^2^—and neck or pharyngo-laryngeal tumors. Two patients with lower scores were managed by elective tracheostomy and awake endoscopic intubation because of local pathology. Caldiroli *et al.* observed Cormack–Lehane grades of III and IV in 0.2% of patients, compared with the 5.8% observed in a large meta-analysis using DL ^5^, and “difficulty” with intubation was encountered in 14 (0.14%) out of 6276. All patients were successfully intubated, although the primary outcome was success rather than the number of attempts or the time required. The study compared outcomes with other publications and thus the patients and care providers may not be comparable. In addition, the study had several significant limitations: it was restricted to neurosurgical procedures, it involved a single relatively small anesthesiology department accustomed to the routine use of the GlideScope, it equated the laryngeal view with the ease of intubation failing to address the number of attempts or amount of time required, and it excluded patients commonly encountered in clinical practice (BMI >30 kg/m ^2^). Nonetheless, this study did show that, in experienced hands, a particular hyperangulated VL (GlideScope) resulted in significantly better laryngeal exposure that ultimately translated into successful tracheal intubation, validating their airway management algorithm.

These novel approaches raise the question of whether it is time to change the “routine approach to laryngoscopy” ^3, 11, 16– 19^, and future studies are needed to confirm the potential benefits of such changes and the optimal ways to prepare and implement such an approach.

The specific type of VL may also play a role. Kleine-Brueggeney *et al*. performed a multicenter randomized controlled trial evaluating six videolaryngoscopes in 720 patients with a simulated difficult airway achieved with a rigid cervical collar ^20^. This reduced mouth opening from a mean of 46 to 23 mm. This trial revealed differences in the performance of the six videolaryngoscopes. The first-attempt success rates were 85 to 98% for all but the A.P. Advance™ difficult airway blade. The highest success and lowest tissue trauma rates were achieved by the McGrath™ using a Macintosh-style blade and C-MAC™ using a D-Blade, highlighting the importance of the VL blade design.

This draws attention to several important points: (i) the design of videolaryngoscopes evolves, and although devices may bear a similar name (for example, McGrath Series 5 and McGrath MAC, GlideScope LoPro, GlideScope Cobalt, and GlideScope T-MAC), performance may differ; (ii) the experience of the laryngoscopist with the device being tested may not be the same as their overall experience as a laryngoscopist and this is not always clear in the publications; (iii) frequently, the patient populations differ, ranging from patients (or manikins) with normal airways, suspected difficult airways, known difficult airways, and simulated difficulty; (iv) the method of simulating a difficult airway (for example, manual inline stabilization or rigid cervical collar) may inadvertently create bias; (v) using a particular device, the larynx may be viewed directly or indirectly ^21^; and (vi) studies may employ or define outcomes differently.

For the reasons stated above, it is very difficult to identify consistent differences in the performance of various VLs, even those similar in design. Aziz *et al*. ^22^ conducted a large multicenter prospective randomized controlled trial and showed that, in patients requiring general anesthesia for elective surgery and presenting with clinical predictors of difficult (direct) laryngoscopy, the C-MAC™ D-Blade, hyperangulated and designed for more challenging airways, did not yield the same first-attempt intubation success as the (hyperangulated) GlideScope. Intubation success rates were high with both devices; the first-attempt success rates were 96.2% and 93.4% with the GlideScope and C-MAC D-Blade, respectively.

In summary, the data from the recent studies suggest that VL as a primary technique in the operating room has the potential to improve intubation-related outcomes. Future research will help us refine optimal approaches to laryngoscopy in the operating room, including patient selection, operator training, and device selection.

### In the intensive care unit

A randomized clinical trial involving 371 adults requiring intubation between 2015 and 2016 in seven ICUs in France compared the McGrath MAC VL (n = 186) with DL (n = 185). The trial showed that intubation using the McGrath MAC provided better glottic exposure but failed to improve the rate of first-attempt success and was associated with higher rates of severe life-threatening complications ^23^. However, important limitations of this study were that intubations were performed by non-experts with limited manikin-based training and stylets were not used. First-attempt success was poor in both groups—126 out of 186 (VL) and 130 out of 185 (DL)—and time to complete intubation was measured in minutes rather than seconds.

A single-center observational study looked at 906 consecutive intubation attempts in the ICU employing VL ^24^. A variety of devices were available, and most intubations were attempted by supervised critical care trainees. Overall, the first-attempt tracheal intubation success rate was 740 (82%) out of 906. The characteristics associated with first-attempt intubation *failure* included the presence of blood in the airway, airway edema, cervical immobility, and obesity. There were insufficient numbers to compare different devices, patient and device selection were at the discretion of the operator, and reasons for failure were determined by the laryngoscopist.

Arulkumaran *et al*. published a systematic review and meta-analysis comparing VL and DL for emergency intubation outside the operating room ^25^. They looked at 32 studies involving over 15,000 emergency intubations. In the ICU, they found that VL (GlideScope and C-MAC) increased first-attempt tracheal intubation success—odds ratio (OR) = 2.02, 95% confidence interval (CI) = 1.43–2.85—and resulted in fewer esophageal intubations, but the benefits of VL were greater for trainees than experienced clinicians. This article followed other reviews that reported conflicting results on the effects of VL use in critically ill patients. The pharmacological management for laryngoscopy and intubation may contribute to success and a reduction of complications ^26, 27^.

A meta-analysis of recent randomized controlled trials comparing VL and DL and restricted to ICU patients analyzed four studies and included 678 patients. Overall, VL did not significantly improve first-attempt tracheal intubation success (relative risk [RR] = 1.17, 95% CI = 0.89–1.53); however, it did improve the laryngeal view and reduced esophageal intubation (RR = 0.31, 95% CI = 0.11–0.90). Furthermore, VL did not reduce the time for successful intubation or severe hypoxia ^28^. As mentioned above, the studies included a variety of devices, some likely to be more effective than others; the intubations were often attempted by trainees and rarely by experts, and all demonstrated low or very low rates of first-attempt success in a patient population at significant risk when multiple attempts are required.

Intubation of critically ill patients differs in several important respects: there may be little time for an adequate airway evaluation, and the patients have reduced physiologic reserves and almost invariably are at increased risk of regurgitation, aspiration, hypoxemia, and hemodynamic instability. Essential equipment and drugs may not be immediately available or familiar, and the support personnel may have less airway management experience. Compared with the elective surgical patient, adverse events relating to airway management in critically-ill patients, are more likely to result in brain injury and death ^29^. Comprehensive guidelines for managing the intubation of critically ill adults have recently been released by three national societies ^29– 31^. They emphasized the importance of human factors such as cognitive overload (“analysis paralysis” ^29^), situational awareness, team integration, and the immediate availability of and familiarity with alternative devices. Preoxygenation with non-invasive ventilation or high-flow nasal oxygen prior to intubation attempts was endorsed to increase apneic tolerance. The Difficult Airway Society ^29^ advocated a “modified” rapid sequence induction, employing gentle mask ventilation and abandonment of cricoid pressure if it hampered laryngoscopy or intubation. Paralytics were encouraged to create conditions that facilitate laryngoscopy, ventilation, and an emergency front-of-neck access should this be required. All of the societies acknowledged the advantages of VL and recommended that a videolaryngoscope should be available and considered as an option for all intubations of critically ill patients ^29– 31^.

In summary, although observational studies have supported the benefit of VL for the outcome of intubation success in the ICU environment, randomized trials have not proven this benefit thus far. Nonetheless, several national guidelines now recommend the immediate availability and, when difficulties are anticipated, primary use of VL in this environment. These recommendations are useful only to the extent that those using the device are appropriately trained.

### In the emergency department

A recent prospective comparison of emergency department tracheal intubations randomly assigned 198 adult patients to undergo either direct viewing (DL) or video viewing (VL) for the first intubation attempt with the Storz C-MAC (Macintosh-style VL). The airways were generally managed by senior residents with at least two years of clinical experience and “extensive” didactic, manikin, and simulation training in the four months prior to the study ^32^. First-attempt success was not significantly different (86% and 92% for DL and VL, respectively), nor were there differences in intubation time, hypoxemia, aspiration, or length of hospital stay. Unfortunately, this study was relatively small and may have been underpowered to detect a difference in first-attempt success, there were numerous protocol violations, and not all physicians felt equally comfortable with both techniques.

The use of adjuncts during laryngoscopy has traditionally been part of clinical practice and guideline recommendations. A randomized single-center study looked at 757 adult patients requiring emergency department intubation with a Macintosh-style VL and compared the use of a coudé-tipped introducer (“bougie”) and a stylet to facilitate initial intubation ^33^. Most of the attempted intubations used the C-MAC, and in the majority of cases, the larynx was viewed directly (rather than using the screen). Driver *et al*. found that, in patients with at least one difficult airway characteristic, the use of a bougie resulted in significantly higher first-attempt intubation success: 96% (CI 93–99%) versus 82% (CI 76–88%) ^33^. Complications were infrequent and not different between the groups. In this study, a significant proportion of patients still had little or no laryngeal exposure (29 out of 191 in the bougie group and 13 out of 182 in the stylet group). A bougie or stylet was attempted even when a Cormack–Lehane IV view was obtained. It is also important to consider that the study took place in a department where operators routinely used a bougie (and not a stylet) for the majority of intubations before initiation of the trial.

A multicenter Japanese study involving 3360 cardiac arrest patients looked at first-attempt intubation success and compared VL and DL ^34^. The study observed an OR of 1.61 (CI 1.26–2.06) favoring VL, better laryngeal exposure, and a lower risk of esophageal intubation ^34^.

In summary, in the emergency department, the use of a coudé-tipped introducer (“bougie”) as an adjunct to DL or Macintosh-style VL may provide significantly higher first-attempt intubation success. Data on the routine use of VL versus DL as a primary technique are conflicting, and future well-designed randomized controlled trials appear necessary to better define this aspect of care.

## Airway assessment and predicting difficulty

The importance of recognizing patients in whom tracheal intubation is likely to be difficult is widely accepted ^35– 38^, although it is obvious that the science lags far behind the need ^5, 39^. Often forgotten ^40^ is that the bedside predictors of difficulty were specifically developed for Macintosh-style DL, although this is rarely stated. These have been shown to have limited relevance to indirect laryngoscopy. Nonetheless, a recent systematic review of high-level studies involving 33,559 patients showed that difficulty (with DL) was encountered in 10% of patients and that the best predictor was the inability to protrude their lower incisors beyond their upper incisors (class 3 “upper lip bite test”; likelihood ratio 14, CI 8.9–22) ^40^. An abnormal upper lip bite test, which is easily assessed at the bedside by clinicians, raises the probability of difficult intubation from 10% to more than 60% for the average-risk patient ^40^. The review concluded that “although several simple clinical findings are useful for predicting a higher likelihood of difficult endotracheal intubation, no clinical finding reliably excludes a difficult intubation” ^40^, making it essential for airway managers to be prepared for failure and able to quickly transition to an alternative technique ^3, 30^.

## Conclusions

Technological advances in laryngoscopy have been introduced into clinical practice in recent years. As we gain a better understanding of such advances, research is focusing on the optimal integration of such technologies in the management of patients in the operating room, ICU, or emergency department. Efforts are focused on optimizing initial laryngoscopy attempts and, when necessary, early transitioning to safe and effective rescue techniques. Concurrently, the use of established techniques remains relevant, and efforts are also devoted to a better understanding of difficult airway prediction and the design of improved airway management algorithms.
